# Alemtuzumab induction therapy in solid organ transplantation

**DOI:** 10.1186/2047-1440-2-S1-S5

**Published:** 2013-11-20

**Authors:** Peter J Friend

**Affiliations:** 1 Nuffield Department of Surgical Sciences, University of Oxford, Oxford Transplant Centre, Churchill Hospital, Oxford OX3 7LJ, UK

**Keywords:** alemtuzumab, induction therapy

## Abstract

Alemtuzumab (Campath) is a monoclonal antibody that has a profound lymphocyte-depleting effect, targeting the CD52 antigen that is present on all lymphocytes. Alemtuzumab has been used for the treatment of chronic lymphocytic leukaemia and various autoimmune disorders, and has also shown potential as an induction agent in the prevention of rejection following solid organ transplantation. Alemtuzumab has been studied in randomised controlled trials and has demonstrated low levels of rejection in renal transplant recipients compared with other induction agents, albeit mainly in the early months following transplantation. Studies have shown that alemtuzumab enables the use of lower calcineurin inhibitor (CNI) maintenance drugs; however, this reduction in nephrotoxic immunosuppression has not consistently been matched by an improvement in renal function. The hypothesis has been suggested that alemtuzumab might allow the development of immunosuppressive regimens that avoid CNIs completely; studies have investigated the combination of alemtuzumab with mammalian target of rapamycin-inhibitor maintenance therapy, and, in particular, sirolimus. Initial studies with this combination showed that regimens of sirolimus alone and of sirolimus with mycophenolate mofetil were unsuccessful, with a high rate of rejection and complications. Subsequent studies have targeted the combination of alemtuzumab induction with a short course of a CNI, before switching to maintenance therapy with sirolimus. This regimen might combine good protection from acute cellular rejection and chronic nephrotoxicity. A randomised controlled trial has been established to study this regimen, with results pending.

## Introduction

The design of an immunosuppressive strategy must take into account the three major causes of morbidity following organ transplantation: ischaemia–reperfusion injury (particularly in marginal donor organs); rejection; and complications of drug treatment (toxicity, infection and cancer). Induction immunosuppression is now used in the majority of transplant programmes in solid organ transplantation.

Induction therapy is intended to protect the graft from the immediate postoperative period of high rejection risk. However, there is increasing interest in the effects of induction agents in mitigating the effects of ischaemia–reperfusion injury. With respect to maintenance therapy, the priority in the early postoperative weeks is to prevent rejection and minimise postsurgical infections and other surgical complications. Subsequently, drug toxicity, opportunistic infection and cancer risk become the primary concerns.

Despite several decades of experience, there is no consensus regarding many aspects of induction therapy. Both polyclonal antibodies and monoclonal antibodies are used with no clear evidence as to which is preferable. Similarly, there is little agreement as to whether depleting or nondepleting antibodies are preferable. There is a range of targets from the highly selective anti-CD25 antibodies (basiliximab) that target an antigen (the inter-leukin-2 receptor) only present on activated lymphocytes to antibodies that recognise all peripheral blood lymphocytes (for example, antithymocyte globulin (ATG), alemtuzumab). Although anti-CD25 antibodies are very widely used globally, there is now increasing interest in the potential benefits of depleting agents with a broader spectrum. The advantages that have been hypothesised for these agents include reduction of ischaemia–reperfusion injury, a more profound protection from early acute rejection and, finally, a long-lasting effect in relation to donor-specific hyporesponsiveness.

## Alemtuzumab

Alemtuzumab (Campath) is a monoclonal antibody that recognises the CD52 antigen, present on all peripheral blood lymphocytes. Campath antibodies have been tested experimentally and clinically for 30 years [1].

Initially an IgM rat-derived antibody (Campath-1M) was used for the removal of T cells from bone marrow for transplantation; this was carried out as a means to prevent graft versus host disease. It was noted at the time that an antibody of profound lymphocyte-depleting capabilities would have potential application in solid organ transplantation. Campath-1M was used in clinical renal transplant trials and was found to have a profound effect on peripheral lymphocyte numbers, although with rapid recovery between doses [2], and was also shown to reduce the incidence of acute rejection [3]. Smaller studies with the IgG2b isotype (Campath-1G) demonstrated a more profound and long-lasting depletion of lymphocytes [4].

The humanised antibody (Campath-1H) was developed with the potential advantages of greater efficacy and, second, a lower risk of sensitisation. This antibody was used for (and subsequently licensed for) the treatment of chronic lymphocytic leukaemia [5]. Campath-1H was also used in a number of autoimmune conditions [6-8] and shown to be effective in treating multiple sclerosis [9]. The first report of the use of the humanised antibody in solid organ transplantation was in the treatment of acute cellular rejection of kidney transplants [10], which was associated with a high incidence of infections.

## Randomised trials of alemtuzumab in solid organ transplantation

A number of randomised trials of alemtuzumab in solid organ transplantation have been published. In 2008 Margreiter and colleagues studied 131 patients randomised to receive induction therapy with alemtuzumab (2 × 20 mg) versus no induction [11]. All patients were subsequently treated with tacrolimus and patients who had not received induction therapy also received mycophenolate. This trial demonstrated a significant reduction in the incidence of acute rejection at 6 months (29% vs. 15%, *P* = 0.05). However, by 12 months the incidence of rejection was no longer significantly different (32% vs. 30%). There was no difference in renal function between the two groups and, apart from an increase in the incidence of cytomegalovirus infection, there was no other adverse consequence of induction therapy.

In 2011 Chan and colleagues reported a randomised controlled trial of 82 alemtuzumab-treated patients (with tacrolimus monotherapy) versus 42 controls (receiving dacluzimab, tacrolimus, mycophenolate), all patients having a rapid steroid taper [12]. This study showed a lower incidence of rejection in the alemtuzumab-treated group at 6 months but not 12 months and no difference in graft survival or function. However, the study confirmed that alemtuzumab therapy does enable simple and effective tacrolimus monotherapy with a very low rate of rejection.

Also in 2011, Hanaway and colleagues carried out a randomised trial involving 474 kidney transplant recipients [13]. These were divided into high-risk patients (defined as panel reactive antibody >20%, black race, re-transplant; *n* = 139) and low-risk patients (all others). The high-risk group was treated with either ATG (1.5 mg/ kg × 4, *n* = 70) or alemtuzumab (30 mg × 1, *n* = 69). The low-risk group of patients was treated with basiliximab (20 mg × 2, *n* = 171) or alemtuzumab (30 mg × 1, *n* = 164). All patients received tacrolimus 8 to 14 ng/ml, mycophenolate 2 g/day, and steroids for 5 days. Patients were followed up for 3 years. This trial demonstrated that patients treated with alemtuzumab had a significantly lower incidence of cellular rejection at 6, 12 and 36 months (3% vs. 15%, 5% vs. 17% and 13% vs. 20% respectively). When divided into the high-risk and low-risk subgroups, it was clear that the reduction in rejection was entirely within the group of patients at low risk of rejection (alemtuzumab vs. basiliximab) and that the incidence of rejection in the high-risk patients was similar between the alemtuzumab and ATG groups. There was no significant difference in graft or patient survival. Analysis of lymphocyte populations demonstrated that alemtuzumab and rabbit ATG had similar effects with respect to lymphocyte depletion and the rate of recovery of lymphocyte populations. This was contrasted in the low-risk group, in which the lymphocyte population was profoundly suppressed by alemtuzumab but very little altered by treatment with basiliximab. There was no effect on renal function. This trial did show that alemtuzumab-treated patients are more liable to late rejection episodes (after 12 months), a finding also noted by others including Watson and colleagues [14]. What is also notable from this and other large series of alemtuzumab-treated transplant patients is the relative absence of autoimmune complications, a problem that has been seen more commonly in patients treated for multiple sclerosis [15].

When the potency and duration of lymphocyte depletion induced by alemtuzumab was recognised, a number of investigators considered whether this may lead to the induction of donor-specific hyporesponsiveness. This followed the important observations of Knechtle and colleagues in demonstrating that profound lymphocyte depletion, using an anti-CD3 monoclonal antibody conjugated to diphtheria toxin, can lead to long-lasting donor-specific hyporesponsiveness in a nonhuman primate experimental model of kidney transplantation [16]. Calne and colleagues tested the use of alemtuzumab induction (2 × 20 mg) followed by low-dose cyclosporin monotherapy (75 to 125 mg/ml) [17]. This study, although without a control group, demonstrated the capability of a low-dose monotherapy immunosuppression regimen to achieve low levels of rejection and good levels of graft survival and function. The same group of patients was reviewed 5 years after transplantation [14] and was compared with a group of matched controls treated with conventional therapy (cyclosporin, prednisolone, azathioprine). Lymphocyte counts were significantly lower in the alemtuzumab group only in the first 3 months postoperatively. Cyclosporin levels were significantly lower in the alemtuzumab group for approximately 2 years. However, despite lower cyclosporin levels, there was no significant difference in renal function. An early benefit with respect to acute rejection in the alemtuzumab-treated patients was matched by an increased level of rejection after 6 months, culminating in a similar overall level of rejection between the two groups. There was no significant difference in patient or graft survival or in the incidence of infection or other serious adverse events.

## Alemtuzumab and mammalian target of rapamycin inhibitors

The ability to lower calcineurin inhibitor (CNI) doses was regarded as one of the primary advantages of alemtuzumab therapy, particularly because of the desire to avoid chronic nephrotoxicity [18]. However, despite the ability of alemtuzumab to enable lower levels of CNI therapy, none of the above studies demonstrated improvement of renal function in patients treated with alemtuzumab. The profound lympho-depletion caused by alemtuzumab led Kirk and colleagues to question whether the antibody alone would enable tolerance and avoid the need for maintenance therapy. Seven living donor recipients were treated with three or four doses of alemtuzumab preoperatively; despite profound depletion of lymphocytes in the peripheral blood and lymph nodes, all patients developed rejection (reversible) and required conversion to maintenance sirolimus [19]. The availability of mammalian target of rapamycin inhibitors, however, provided the opportunity for long-term maintenance of patients without the use of nephrotoxic immunosuppression; the combination of alemtuzumab induction with sirolimus monotherapy was a theoretically attractive proposition.

In the first study of an immunosuppression strategy of this design, 29 patients were treated with alemtuzumab (20 mg × 2) followed by low-dose maintenance sirolimus therapy [20]. Eight patients required treatment for rejection and one graft was lost. The investigators concluded that sirolimus monotherapy was inadequate in this context. Other investigators tested the combination of sirolimus with mycophenolate following alemtuzumab induction [21]. Twenty-two patients were treated with alemtuzumab induction (30 mg × 2) and maintenance therapy sirolimus (8 to 12 mg/ml) and mycophenolate (500 mg/twice daily). There was an acute rejection incidence of 36%, leukopaenia in 27% of patients and acute respiratory distress syndrome in two patients. The investigators concluded that the combination of alemtuzumab, sirolimus and mycophenolate, when started at the time of transplantation, was associated with a high rejection rate and a high incidence of other complications.

Attention then moved to the use of alemtuzumab induction followed by a short course of CNI therapy, before switching to sirolimus maintenance therapy for the longer term. In a study of 30 patients, alemtuzumab (30 mg × 2) induction was followed by mycophenolate (500 mg/twice daily for 12 months), tacrolimus (5 to 8 ng/ml for 6 months) and, after tacrolimus withdrawal, sirolimus (5 to 8 ng/ml) (data submitted for publication). A number of rejection episodes occurred following mycophenolate withdrawal at 12 months (all reversible with steroids) and the protocol was therefore amended to continue mycophenolate 250 mg twice daily thereafter. This protocol was associated with a low incidence of rejection (6.6% at 12 months). Following the change of protocol to maintain low-dose mycophenolate after 12 months, there were no acute rejection episodes after 12 months. Two patients were withdrawn from the protocol because of respiratory side effects of sirolimus. Eighty-five per cent of patients remained steroid and CNI free 5 years postoperatively.

This trial demonstrated the potential utility of alemtuzumab in enabling patients to be established safely on CNI-free therapy. The real benefit of this strategy is in the potential absence of chronic nephrotoxicity and the consequent long-term improvement in graft half-life. Clearly this hypothesis required formal testing in an adequately powered randomised control clinical trial. Such a trial was established in 2010 with the intention of testing the effects of alemtuzumab versus basiliximab as an induction agent and testing the effect of switching to sirolimus from tacrolimus at 6 months. The end points for this study will first be the incidence of acute cellular rejection and then medium-term graft function. The results of this study (Campath, calcineurin inhibitor reduction, chronic allograft nephropathy) are awaited.

## Abbreviations

ATG: antithymocyte globulin; CNI: calcineurin inhibitor.

## Competing interests

PJF has received honoraria from Pfizer for lecturing.

## Declaration

This article has been published as part of *Transplantation Research* Volume 2 Suppl 1, 2013: Proceedings of the 12th International Symposium on Transplantation. The full contents of the supplement are available at http://www.transplantationresearch.com/supplements/2/S1. The supplement is based on the 12th International Transplantation Symposium held on 16–17 April 2012, in Istanbul, Turkey. The symposium and publication based on these proceedings were supported by an unrestricted educational grant from Pfizer Inc., who had no influence on the editorial content. Editorial support was provided by Synergy, Richmond, UK and was funded by Pfizer Inc. Rapamune (sirolimus) is a product marketed by Pfizer Inc. The articles have been through the journal’s standard peer review process and the Supplement Editors declare that they have no competing interests.

## References

[B1] Hale G Bright S Chumbley G Hoang T Metcalf D Munro AJ Waldmann H Removal of T cells from bone marrow for transplantation: a monoclonal antilymphocyte antibody that fixes human complement Blood 1983 2 873 882 6349718

[B2] Hale G Waldmann H Friend P Calne R Pilot study of CAMPATH-1, a rat monoclonal antibody that fixes human complement, as an immunosuppressant in organ transplantation Transplantation 1986 2 308 311 10.1097/00007890-198609000-00017 3529531

[B3] Friend PJ Hale G Waldmann H Gore S Thiru S Joysey V Evans DB Calne RY Campath-1M-prophylactic use after kidney transplantation. A randomised controlled clinical trial Transplantation 1989 2 248 253 10.1097/00007890-198908000-00013 2667209

[B4] Friend PJ Waldmann H Hale G Cobbold S Rebello P Thiru S Jamieson NV Johnston PS Calne RY Reversal of allograft rejection using the monoclonal antibody, Campath-1G Transplant Proc 1991 2 2253 2254 1908152

[B5] Lundin J Kimby E Björkholm M Broliden PA Celsing F Hjalmar V Möllgård L Rebello P Hale G Waldmann H Mellstedt H Osterborg A Phase II trial of subcutaneous anti-CD52 monoclonal antibody alemtuzumab (Campath-1H) as first-line treatment for patients with B-cell chronic lymphocytic leukemia (B-CLL) Blood 2002 2 768 773 10.1182/blood-2002-01-0159 12130484

[B6] Isaacs JD Manna VK Rapson N Bulpitt KJ Hazleman BL Matteson EL St Clair EW Schnitzer TJ Johnston JM CAMPATH-1H in rheumatoid arthritis – an intravenous dose-ranging study Br J Rheumatol 1996 2 231 240 10.1093/rheumatology/35.3.231 8620297

[B7] Dick AD Meyer P James T Forrester JV Hale G Waldmann H Isaacs JD Campath-1H therapy in refractory ocular inflammatory disease Br J Ophthalmol 2000 2 107 109 10.1136/bjo.84.1.107 10611109 PMC1723242

[B8] Cheung WW Hwang GY Tse E Kwong YL Alemtuzumab induced complete remission of autoimmune hemolytic anemia refractory to corticosteroids, splenectomy and rituximab Haematologica 2006 2 5 Suppl ECR13 16709521

[B9] Moreau T Thorpe J Miller D Moseley I Hale G Waldmann H Clayton D Wing M Scolding N Compston A Preliminary evidence from magnetic resonance imaging for reduction in disease activity after lymphocyte depletion in multiple sclerosis Lancet 1994 2 298 301 10.1016/S0140-6736(94)91339-0 7914262

[B10] Friend PJ Rebello P Oliveira D Manna V Cobbold SP Hale G Jamieson NV Jamieson I Calne RY Harris DT Successful treatment of renal allograft rejection with a humanized antilymphocyte monoclonal antibody Transplant Proc 1995 2 869 870 7879212

[B11] Margreiter R Klempnauer J Neuhaus P Muehlbacher F Boesmueller C Calne RY Alemtuzumab (Campath-1H) and tacrolimus monotherapy after renal transplantation: results of a prospective randomized trial Am J Transplant 2008 2 1480 1485 10.1111/j.1600-6143.2008.02273.x 18510632

[B12] Chan K Taube D Roufosse C Cook T Brookes P Goodall D Galliford J Cairns T Dorling A Duncan N Hakim N Palmer A Papalois V Warrens AN Willicombe M McLean AG Kidney transplantation with minimized maintenance: alemtuzumab induction with tacrolimus monotherapy – an open label, randomized trial Transplantation 2011 2 774 780 10.1097/TP.0b013e31822ca7ca 21836540

[B13] Hanaway MJ Woodle ES Mulgaonkar S Peddi VR Kaufman DB First MR Cray R Holman J INTAC Study Group Alemtuzumab induction in renal transplantation N Engl J Med 2011 2 1909 1919 10.1056/NEJMoa1009546 21591943

[B14] Watson CJ Bradley JA Friend PJ Firth J Taylor G Bradley JR Smith KG Thiru S Jamieson NV Hale G Waldmann H Calne R Alemtuzumab (CAMPATH 1H) induction therapy in cadaveric kidney transplantation – efficacy and safety at five years Am J Transplant 2005 2 1347 1353 10.1111/j.1600-6143.2005.00822.x 15888040

[B15] Coles AJ Compston DA Selmaj KW Lake SL Moran S Margolin DH Norris K Tandon PK Alemtuzumab vs. interferon beta-1a in early multiple sclerosis N Engl J Med 2008 2 1786 1801 18946064 10.1056/NEJMoa0802670

[B16] Knechtle SJ Vargo D Fechner J Zhai Y Wang J Hanaway MJ Scharff J Hu H Knapp L Watkins D Neville DM Jr FN18-CRM9 immunotoxin promotes tolerance in primate renal allografts Transplantation 1997 2 1 6 10.1097/00007890-199701150-00002 9000652

[B17] Calne R Friend P Moffatt S Bradley A Hale G Firth J Bradley J Smith K Waldmann H Prope tolerance, perioperative Campath 1H, and low-dose cyclosporin monotherapy in renal allograft recipients Lancet 1998 2 1701 1702 9734890 10.1016/S0140-6736(05)77739-4

[B18] Nankivell BJ Borrows RJ Fung CL O’Connell PJ Allen RD Chapman JR The natural history of chronic allograft nephropathy N Engl J Med 2003 2 2326 2333 10.1056/NEJMoa020009 14668458

[B19] Kirk AD Hale DA Mannon RB Kleiner DE Hoffmann SC Kampen RL Cendales LK Tadaki DK Harlan DM Swanson SJ Results from a human renal allograft tolerance trial evaluating the humanized CD52-specific monoclonal antibody alemtuzumab (CAMPATH-1H) Transplantation 2003 2 120 129 10.1097/01.TP.0000071362.99021.D9 12865797

[B20] Knechtle SJ Pirsch JD H Fechner J Jr Becker BN Friedl A Colvin RB Lebeck LK Chin LT Becker YT Odorico JS D’Alessandro AM Kalayoglu M Hamawy MM Hu H Bloom DD Sollinger HW Campath-1H induction plus rapamycin monotherapy for renal transplantation: results of a pilot study Am J Transplant 2003 2 722 730 10.1034/j.1600-6143.2003.00120.x 12780564

[B21] Flechner SM Friend PJ Brockmann J Ismail HR Zilvetti M Goldfarb D Modlin C Mastroianni B Savas K Devaney A Simmonds M Cook DJ Alemtuzumab induction and sirolimus plus mycophenolate mofetil maintenance for CNI and steroid-free kidney transplant immunosuppression Am J Transplant 2005 2 3009 3014 10.1111/j.1600-6143.2005.01123.x 16303017

